# Locus of control as a modifiable risk factor for cognitive function in midlife

**DOI:** 10.18632/aging.101490

**Published:** 2018-07-12

**Authors:** Emma Anderson, Alice Cochrane, Jean Golding, Stephen Nowicki

**Affiliations:** 1 MRC Integrative Epidemiology Unit at the University of Bristol, Bristol BS8 2BN, UK; 2 Centre for Child and Adolescent Health, Bristol Medical School, University of Bristol, Oakfield House, Oakfield Grove, Bristol BS8 2BN, UK; 3 Department of Psychology, PAIS Building, Emory University, Atlanta, GA 30322, USA

**Keywords:** cognitive decline, risk factor, internality, externality, ALSPAC

## Abstract

Few modifiable risk factors for cognitive decline have been identified. Despite an external locus of control (LoC) being adversely associated with many psychological and physical health outcomes, few studies have examined whether it is related to cognitive function in adulthood. In 1178 women from the Avon Longitudinal Study of Parents and Children, we examined whether LoC, and change in LoC over time, is associated with cognitive function in midlife. LoC was prospectively measured at mean ages 30 and 48 years using the validated Nowicki-Strickland scale. Cognitive function was examined at mean age 51 years. At both time points, greater externality was associated with lower cognitive function. For example, the group of women classified as being external at mean age 48 years had, on average, a 0.18 lower cognitive function score (95% CI: (0.11 to 0.25) than the group classified as being internal (p<0.001). Participants who changed from external to internal over time, on average, had better cognitive function than those who remained external or changed to become external. In summary, an external LoC may be detrimental to cognitive function. Thus, interventions to increase internality may help to minimise the adverse consequences on cognitive health later in life.

## Introduction

Locus of control (LoC) is defined as the degree to which persons expect that outcomes are contingent on their own behaviour or personal characteristics, as opposed to a function of chance, luck, fate, under control of powerful others, or simply unpredictable [1]. Individuals with a strong internal LoC tend to believe events in their life are primarily a result their own actions. Conversely, people with a strong external LoC tend to believe events in their life are primarily a result of external factors (e.g. fate or luck). LoC is an important personality construct that has been associated with many psychological [2–6] and physical [7,8] health outcomes as well as health behaviours such as smoking and alcohol consumption [9–12]. Studies generally report better outcomes in individuals classified as being more internal than external, concluding that it is psychologically healthy to perceive that one has control over those things which one is capable of influencing. LoC is not static; studies have shown that it is malleable in response to both life events (such as trauma [13,14]) and interventions such as cognitive training [15–17], thus suggesting that it is possible to improve a person’s ability to internalise, which may incur later health benefits.

Despite many psychological and physical health outcomes being studied in relation to LoC [5,7], studies examining whether LoC is related to cognitive function are scarce. Most studies to date have focused on whether cognitive training is associated with short-term [18] and long-term [16] changes in LoC (i.e. the opposite direction). Those existing studies that have examined whether LoC is associated with later cognitive function have mainly been conducted in very small sample sizes [19] or in elderly populations [20,21], whereby reverse causation (i.e. impairment of, or decline in, cognitive function affecting LoC) cannot be ruled out. Wight et al reported that an external LoC was associated with lower cognitive function in a community sample of 1835 men aged 45-59 years from the United States [22]. However, to our knowledge, no studies have examined the association in women. Given that there is evidence to suggest LoC is intervenable, it would be useful to ascertain whether individuals with an internal LoC have greater cognitive function, particularly in later life, as this may help individuals to maintain independence into old age and even potentially reduce risk of cognitive decline and dementia. Current evidence for modifiable risk factors for lower cognitive function in later life is scarce, thus, trying to identify factors on which we can intervene is crucial for prevention strategies.

In a general population sample of British women from the Avon and Longitudinal Study of Parents And Children, we aimed to examine whether (i) LoC at two time-points during adulthood (mean ages 30 and 48 years) and (ii) change in LoC across these two time-points, is prospectively associated with a composite measure of overall cognitive function at mean age 51 years, or with specific cognitive domains including long- and short-term memory, verbal fluency and intelligence, and processing speed.

## RESULTS

### Descriptive characteristics of the participants

There was evidence that women included in these analyses, on average, were more likely to be white compared to non-white, to have a higher SEP and be classified as having an internal LoC compared with women excluded due to missing data (Table 1). However, the magnitude of the differences for ethnicity and SEP was small, and the mean LoC scores at both time points were similar in included versus excluded women. Correlations between the LoC scores at the two time-points was moderate (Pearson’s R=0.53). Approximately 30% of participants changed their LOC over time (12% of participants changed from external to internal and 18% changed from internal to external). Correlations between each of the cognitive function measures (Table S1) were weak to moderate: Pearson’s *r* ranged from 0.15 to 0.43. Logical memory and delayed logical memory were strongly correlated (*r*=0.83).

**Table 1:** Characteristics of participants.

	**Included participants (n=1178)**		**Excluded participants (n=1179) ^†^**	
	**Mean (SD)/** **Percentage (%)**		**N with available data**	**Mean (SD)/** **Percentage (%)**
**Exposures**				
***Mean age 30 years***				
Continuous locus of control score at mean age 30 years	3.30 (1.86)		1,156	3.87 (2.02)
Categorical locus of control at age 30 years (%)^‡^			1156	
Internal	56.20			46.19
External	43.80			53.81
***Mean age 48 years***				
Continuous locus of control score at mean age 48 years	3.03 (1.87)		784	3.58 (2.01)
Categorical locus of control at age 48 years (%)^‡^			784	
Internal	63.07			51.91
External	36.93			48.09
***Change in locus of control score from 30 to 48 years (%)***				
Internal to Internal	44.65		523	27.15
External to External	25.38			37.86
Internal to External	11.54			15.68
External to Internal	18.42			19.37
**Outcome**				
Cognitive function score	4.07 (0.59)		1,654	3.91 (0.61)
**Covariables**				
Age at outcome assessment (years)	50.96 (4.39)		1779	50.74 (4.42)
Ethnicity (%)			1558	
White	98.56			96.79
Non-white	1.44			3.21
Highest qualification (%)			1022	
CSE	34.30			38.65
Vocational	22.16			21.14
O-level	17.15			14.09
A-level	19.86			20.06
Degree	6.54			6.07
Head of household social class (%)			1202	
Professional	24.45			17.80
Managerial and technical	48.73			49.92
Skilled non-manual	20.97			24.96
Non-skilled manual	4.75			5.74
Partly and unskilled manual	1.10			1.58

SD – standard deviation

^†^Excluded participants are those who were eligible to be included in the analysis (i.e. they attended the outcome assessment clinic) but were excluded due to missing data for one LoC measure and one or more potential confounders

^‡^Participants scoring below or equal to the median (3 points) on the locus of control questionnaire were categorised as being internal. Those scoring above the median were categorised as being external.

### Associations between the continuous LoC scores and overall cognitive function

Table 2 shows associations of the continuous LoC scores at the two time-points with the composite measure of cognitive function. There was evidence that a greater LoC score (i.e. greater externality) measured at both time-points (i.e. mean ages 30 and 48 years) was associated with a lower cognitive function score at mean age 51 years, with the magnitude of the association being similar for both time-points. For example, per one-unit increase in LoC score at mean age 48 years, cognitive function scores were, on average, 0.06 lower (95% CI: -0.08 to -0.04), even after adjusting for educational attainment, head of household social class, ethnicity and age at outcome assessment and the previous LoC at mean age 30 years (p<0.001).

**Table 2:** Associations of the continuous locus of control score at two time-points with the composite cognitive function score at mean age 51 years (N=1178).

	**Unadjusted**			**Adjusted for potential confounders^†^**		**Additionally adjusted for earlier locus of control score**	
	**Beta (95% CI)**	**P**		**Beta (95% CI)**	**P**	**Beta (95% CI)**	**P**
	-0.08 (-0.10 to -0.06)	<0.001		-0.07 (-0.08 to -0.05)	<0.001	-	-
							
**Locus of control score at mean age 48 years**	-0.09 (-0.11 to -0.08)	<0.001		-0.06 (-0.08 to -0.04)	<0.001	-0.06 (-0.08 -0.04)	<0.001

CI- confidence interval. Results are interpreted as the average difference in the composite cognitive function score per unit increase in the locus of control score

^†^Adjusted for educational attainment, head of household social class, ethnicity and age at outcome assessment

### Associations between categorical LoC (internal vs external) and overall cognitive function

There was evidence that participants who were categorised as having an external LoC had, on average, lower cognitive function than those who were categorised as being internal, and the magnitude of association was similar at both LoC measurement occasions (Table 3). For example, the external group at mean age 48 years had, on average, a 0.18 lower cognitive function score (95% CI: -0.25 to -0.11) than the internal group (p<0.001), even after adjusting for educational attainment, head of household social class, ethnicity and age at outcome assessment and LoC score at mean age 30 years.

**Table 3:** Average difference in the composite cognitive function score at mean age 51 years between participants who were categorised as having an external vs internal locus of control at two time-points (N=1178).

	**Unadjusted**			**Adjusted for potential confounders^†^**		**Additionally adjusted for earlier locus of control score**	
	**Mean difference in cognitive function score (95% CI)**	**P**		**Mean difference in cognitive function score (95% CI)**	**P**	**Mean difference in cognitive function score (95% CI)**	**P**
**External vs internal locus of control at mean age 30 years**	-0.28 (-0.35 to -0.22)	<0.001		-0.22 (-0.29 to -0.16)	<0.001	-	-
							
**External vs internal locus of control at mean age 48 years**	-0.30 (-0.37 to -0.24)	<0.001		-0.25 (-0.32 to -0.19)	<0.001	-0.18 (-0.25 to -0.11)	<0.001

CI- confidence interval. Results are interpreted as the average difference in the composite cognitive function score between external versus internal locus of control

^†^Adjusted for educational attainment, head of household social class, ethnicity and age at outcome assessment

### Associations between change in LoC over time and overall cognitive function

On average, individuals had the lowest cognitive function scores when they were classified as having an external LoC at both time-points (Table 4). Participants classified as having an external LoC at any of the measurement occasions had lower cognitive function scores than those who sustained an internal LoC over time. Finally, participants who changed from external to internal, on average, had higher cognitive function scores than those who remained external or changed from internal to external.

**Table 4:** Associations between change in locus of control from mean age 30 years to 48 years and the composite cognitive function score at mean age 51 years (N=1178).

		**Unadjusted**			**Adjusted for potential confounders^†^**	
	**Percentage (N) participants**	**Mean difference in cognitive function score (95% CI)**	**P**		**Mean difference in cognitive function score (95% CI)**	**P**
**Change in LoC**						
Internal to internal (reference group)	44.7 (526)	-	-		-	-
External to external	25.4 (299)	-0.43 (-0.51 to -0.35)	<0.001		-0.35 (-0.43 to -0.27)	<0.001
Internal to external	11.5 (136)	-0.20 (-0.31 to -0.10)	<0.001		-0.18 (-0.28 to -0.07)	<0.01
External to internal	18.4 (217)	-0.18 (-0.27 to -0.09)	<0.001		-0.14 (-0.23 to -0.05)	<0.01

CI- confidence interval. Results are interpreted as the average difference in the composite cognitive function score between each group compared to the reference group.

^†^Adjusted for educational attainment, head of household social class, ethnicity and age at outcome assessment

### Additional analyses

Participants classified as having an external LoC had, on average, lower scores for each of the six cognitive tests than those classified as internal (Table S2). The magnitude of association was very similar for each of the tests and across the two time-points that LoC was measured. On average, individuals had the lowest scores on each of the six cognitive tests when they were classified as having an external LoC at both time-points (Table S3). Participants classified as having an external LoC at any of the measurement occasions had lower cognitive scores for each of the six cognitive tests than those who sustained an internal LoC over time and again, the magnitude of association was very similar for each test. Unadjusted associations looked very similar in the larger group of participants with a measure of LoC at the first-time point only (n=2241, Table S4) compared to the results from the main analysis sample (n=1178). Results also looked very similar when additionally adjusted for exposure to psychosocial adversity in childhood as a potential confounder (n=929, Table S5).

## DISCUSSION

We found evidence that having a more internal locus of control in early and mid-adulthood is prospectively associated with better cognitive function, and importantly, that changing to have an internal LoC over the duration of the study was associated with better cognitive function than remaining external or changing to from internal to external. These findings suggest that LoC may be an amenable target for interventions aimed at increasing internality, to improve cognitive function in adulthood and consequently, help to reduce risk of ageing-related morbidity (including Alzheimer’s disease, which has been consistently associated with lower cognitive function in mid-life and mortality [23,24]). Several studies have provided evidence that LoC is modifiable, with cognitive training interventions increasing internality [15–17], and Nowicki et al. [25] have identified several factors associated with changes towards both internality and externality. Our study identified a subgroup of participants (~30%) whose LoC changed over time (i.e. internal to external and vice versa), supporting the assumption that it is not a static construct and may be amenable to intervention. Given that lower cognitive function in adulthood is associated with a lack of functional dependence in old age [26–28], greater risk of cognitive decline and dementia [23,24], and higher mortality rates [29–32], identifying modifiable risk factors is important for informing prevention strategies.

We found evidence using both the continuous LoC score and the categorical (external vs internal) variable, suggesting that LoC across the whole spectrum is related to cognitive decline and that there is not likely to be a threshold effect whereby a certain degree of externality is detrimental for cognitive functioning. We also assessed associations of LoC with individual cognitive tests, as well as a composite score of overall cognitive function. Different cognitive tests measure different underlying systems (e.g. fluid vs crystallised intelligence) and assessing them individually may help identify possible underlying mechanisms of association. Combining measures into a composite score may, however, increase power because summing them together identifies a much higher risk group (i.e. those performing very badly on all tests), which may drive associations. In our study, higher LoC scores (i.e. greater externality) were associated with poorer performance on all individual cognitive tests, with a similar magnitude of effect for each. In addition, change in LoC overtime was similarly associated with each of the cognitive tests, suggesting that there is not one particular aspect of cognition that is largely affected by LoC, but that it influences all domains of cognitive function including memory, processing speed and verbal fluency.

### Comparisons with other studies

Although there is a large body of evidence showing LoC to be associated with many psychological [2,3,5,6,33] and physical [7,8] health outcomes and health behaviours such as smoking [9–12] and alcohol consumption [9,17], studies examining whether LoC is related to cognitive function are scarce [22]. Several studies to date have assessed whether cognitive training is associated with short-term [18] and long-term [16] changes in LoC (i.e. examining the question in the opposite direction) and the findings suggest the relationship may be bidirectional in nature. The few existing studies that have examined whether LoC is associated with later cognitive function been conducted in very small sample sizes (N=<350) [19] or in elderly populations [20,21] where it is impossible to rule out reverse causation (i.e. age-related changes in cognitive function affecting LoC). One study previously reported that an external LoC was associated with lower cognitive function in men from the United States [22]. Converse to our study, the authors found LoC to be very stable over time (LoC changed over an 18-year follow-up period in 30% of our study participants). Similar to our study however, they reported cognitive function to be highest among men who consistently demonstrated an internal locus of control over time, and lowest among those who demonstrated an external propensity over time. Our findings suggest that associations are similar in women and that they can be observed as early as age ~50 years. In addition to this, another study reported that mothers with an internal prenatal LoC, on average, had offspring with a higher IQ at age 8 years [34], suggesting that LoC may be associated with cognitive function across the entire life course; not just in mid-late adulthood.

### Strengths and limitations

To the best of our knowledge, this is the first study to assess associations between LoC measures and cognitive function in women from the general population. LoC was measured using a validated questionnaire [35] at two time-points, meaning we were able to examine how change in LoC relates to our outcome of interest. We also had data for a variety of cognitive tests, enabling us to assess the effect of LoC on different cognitive domains and also on overall cognitive functioning. One limitation of our study is the possibility of selection bias, as 41% of the people who attended the follow-up clinic where the cognitive tests were conducted did not have data for one or more measures of LoC and/or one or more potential confounders. To examine the possibility of selection bias, we examined whether the unadjusted associations were similar in a larger sample of participants with only a measure of LoC at the first-time point and a measure of cognitive function (n=2241 compared to n=1178 in our main analyses). Results were very similar, suggesting that selection bias is unlikely to fully explain our findings. It is also worth noting that people with an external locus of control and with lower cognitive function scores are more likely to be lost to follow-up, meaning any bias due to selection is likely to be towards the null. Finally, we only studied British women, thus we cannot assume that our results would generalise to men, or women from different national or ethnic backgrounds.

## CONCLUSION

Our findings suggest that an external LoC is detrimental to cognitive function. LoC can change over time, and interventions to increase internality may help to minimise the adverse consequences on cognitive health later in life. Further longitudinal studies should examine whether LoC is associated with cognitive function across the whole life course and with cognitive decline over time. Genetic studies may be able to identify variants associated with LoC, which would pave the way for examining whether or not these associations are causal, using methods such as Mendelian randomization.

## MATERIALS AND METHODS

### Study population

The Avon Longitudinal Study of Parents and Children (ALSPAC) is a prospective birth cohort study from southwest England that recruited 14,541 pregnant women, resident in 3 Bristol-based health districts, with an expected date of delivery between April 1991 and December 1992 [43] . Our analysis uses data from the mothers in this cohort [36]. The study website contains details of all available data through a fully searchable data dictionary and variable search tool (www.bris.ac.uk/alspac/researchers/our-data/). Ethical approval for the study was obtained from the ALSPAC Ethics and Law Committee and the Local Research Ethics Committees. Approximately 25 years after recruitment into the cohort, women were invited to attend a follow-up research clinic at which cognitive function was assessed. A total of 2893 woman attended this clinic (mean age 51 years, standard deviation 4.4 years).

### Assessing Locus of Control

Women completed a condensed version of the Adult Nowicki Strickland Internal External control scale (ANSIE) [35] in questionnaires administered at mean ages 30 and 48 years. The original ANSIE comprises 40 items in a yes/no format, which assess perceived control. The version used in the present study comprises 12 of the original 40 items, which were chosen after factor analysis of the ANSIE administered as a pilot to 135 mothers [34]. The 12 questions loaded onto a single factor of general LoC. The 12 questions used are shown elsewhere (Golding et al., 2017). From the responses, a ‘LoC score’ was derived; the higher the score the more external the LoC. Scores ranged from 0 to 12 and approximated a normal distribution, with a median of 3 at both measurement occasions.

### Assessing cognitive function in mid-adulthood

A series of tests were conducted at a follow-up research clinic at mean age 51 years to assess different domains of cognitive function including verbal fluency, short-term and prospective memory and processing speed. All cognitive function outcomes measured in this study are associated with mortality [37]. Cognitive function was assessed with verbal fluency [38], logical memory [39], delayed logical memory [39], digit backwards [40], digit symbol coding [40], and spot the word tests [41]. Full assessment details of each cognitive test are provided in the online Supplement.

### Covariables

We considered social class and ethnicity to be potential confounders of the association between LoC and cognitive function in mid-adulthood. Participants’ social class was indicated by both head of household social class and educational attainment. Participants’ SEP in adulthood was reported at enrolment into the study (during years 1991-1992) as the highest of own and partner’s occupational class groups using the 1991 British Office of Population and Census Statistics (OPCS) classification. It was coded as ‘professional’, ‘managerial and technical’, ‘skilled non-manual’, ‘skilled manual’ and ‘partly or unskilled manual’. Women reported their ethnicity in questionnaires administered at enrolment. Age at the time of outcome assessment was recorded.

### Statistical analysis

### *Locus of control measures*

We examined associations using both a continuous and categorical measure of LoC at mean ages 30 and 48 years. This enabled us to examine whether associations exist across the whole spectrum of LoC or whether there is likely a threshold effect. The continuous scores ranged from 0 to 12 and were normally distributed with a mean (standard deviation, SD) of 3.3 (1.9) at age 30 years and 3.0 (1.9) at age 48 years. The higher the score the more external the locus of control. The categorical measures classify participants as having an internal or external LoC at each time-point, with external being defined as above the median score of 3 (on both measurement occasions), and internal equal to or lower than the median score. Finally, we generated a measure of change in LoC across the two time-points which consisted of four categories: (1) external at both time-points, (2) internal at both time-points, (3) internal to external and (4) external to internal.

### *Generating composite scores of cognitive function*

In addition to assessing individual cognitive function tests which reflect different underlying systems (e.g. fluid vs crystallised intelligence), composite scores of cognitive function were also created using the method devised by Guralnik et al [42]. Combining measures into a composite score may identify a much higher risk group (i.e. participants doing very badly on all tests), thus allowing us to assess the extremes of cognitive performance, which may be more revealing in a middle-aged population that is generally functioning well. Each cognitive function test score was rescaled to lie between 0 and 1, giving all measures equal weight in the final composite scores (see online Supplement for further details of the rescaling procedure). Participants unable to perform a test were assigned a value of 0. Rescaled cognitive function measures were summed to create normally distributed aggregate cognitive function score with a range of 0 to 6.

### *Examining associations between LoC and cognitive function*

We examined associations of the continuous LoC scores, the categorical LoC measure and the change in LoC measure with the composite measure of overall cognitive function. All associations were examined in the following models: 1) unadjusted, 2) adjusted for educational attainment, head of household social class, ethnicity and age at outcome assessment and 3) additionally adjusting for the previous measure of LoC (except for where change in LoC is the exposure).

### *Eligibility criteria and missing data*

Participants were eligible to be included in analyses if they had data for all variables included in the analyses (i.e. all six cognitive function tests, measures of LoC at mean ages 30 and 48 years and all potential confounders, n=1178). A total of 1779 women were excluded from these analyses due to missing data for one or more of these variables. To investigate potential selection bias due to missing data, we examined whether unadjusted associations were similar in the larger sample of participants with a measure of LoC at the first-time point only (i.e. at mean age 30 years, n=2241).

### Additional analyses

Firstly, we examined associations of the continuous LoC scores and change in LoC with the six individual cognitive tests, to establish whether LoC (or change in LoC) is particularly strongly associated with certain cognitive domains. Secondly, we considered that exposure to psychosocial adversity (for example, physical or emotional abuse and neglect) in childhood may be a potential confounder of the association between LoC and cognitive function. However, only 79% of participants additionally had a measure of exposure to psychosocial adversity in childhood. Thus, adjusting for this in our main analysis would have greatly reduced our sample size (and thereby statistical power). To examine whether associations were likely to be confounded by exposure to psychosocial adversity, we assessed whether associations between LoC and cognitive function were similar after additional adjustment for psychosocial adversity in the subgroup of participants with data for this variable (n=929). Details of the measure of psychosocial adversity are provided in the online Supplement.

## Supplementary Material

Supplementary File
