# Supplementary material for: Locus of control as a modifiable risk factor for cognitive function in midlife
Source: Aging (Albany NY). 2018 Jul 12;10(7):1542–55. doi: 10.18632/aging.101490 (PMC6075438; doi:10.18632/aging.101490)
Supplement: Supplementary File [file aging-10-101490-s001.pdf]

SUPPLEMENTARY MATERIAL

SUPPLEMENTARY METHODS

Assessing cognitive function

To assess logical memory, a standardised recording of a short story was played to the participant. They were asked to listen carefully and try to remember it the way it was said. After playing the story, the participant was asked to tell the fieldworker everything they could remember about the story, starting at the beginning. One point was scored for each correct item recalled according to standard scoring guidelines. To assess delayed logical memory, the participant was asked to recall the story again after completing all other cognitive tests. To assess backwards digit span, the fieldworker gave a series of numbers out loud and asked the participant to recall them backwards with no time for pause. There were two practise items and seven test items (each item having two trials). Each item had one more number to recall than the preceding item (item 1 had two digits, item 7 had eight digits). The test was discontinued if a participant scored 0 on both trials of any item. In the spot-the-word test the participant was given a series of sixty pairs of words. Each pair contained one real and one nonsense word. The participant was asked to place a tick next to the word in each pair that they thought was the real word. The participant was scored 1 point for each correct answer. In the digit symbol coding test the participant was shown a series of symbols, each one associated with the numbers 1 to 9. They were then asked to fill in a grid drawing the correct symbol next to each number one after the other without skipping any. The task was timed for 120 seconds. The participant was scored 1 point for each correct entry done in the allocated time. To assess verbal fluency, the participant was given a letter of the alphabet (C, F and L) and asked to say as many words as they can think of that beginning with that letter. They were not allowed to include proper nouns or include the same word with a different ending. A practise was given with the letter 'S', after which, 1-minute periods were timed for the letters 'C', 'F' and 'L'. The participant scored one point for each correct entry done in the allocated time with a total score obtained as the sum of all three scores. Measure was converted to speed in meters per second.

Rescaling the cognitive function measures

Participants with test scores above the 99th percentile were coded to the 99th percentile value. Participant scores were then divided by the 99th percentile value give a value between 0 and 1, with one being equal to the 99th percentile score. All rescaled measures were coded in the same direction so that 0 represents the poorest performance and 1 represents highest performance (i.e. the 99th percentile). Participants unable to perform each test for health reasons were assigned a value of 0. The six rescaled cognitive function test scores were summed to create a normally distributed aggregate score ranging from 0 to 6.

Assessing exposure to psychosocial adversity

Women retrospectively reported childhood psychosocial adversity in questionnaires administered at the time of enrolment into the study, throughout pregnancy and postnatally (from 12 weeks gestation to 33 months postnatally, mean ages at the time of reporting ranged between 28 to 30 years). *A priori,* we aimed to examine the same adversity measures as the Adverse Childhood Experiences (ACE) study. However, ALSPAC measured many additional forms of adversity to this study. Thus, we decided to include as many types of psychosocial adversity as possible.

The following forms of psychosocial adversity were assessed in the questionnaires: maternal lack of care and maternal overprotection, maladaptive family functioning, parental mental illness, sexual abuse and non-sexual abuse. Questions about maternal care and overprotection were based on a validated instrument for assessing maternal bonding.(Parker, 1990) Maladaptive family functioning includes questions that assess the nature of the relationship between the participant's mother and father (i.e. whether the relationship was, for example, stable and predictable, affectionate, violent, respectful). Parental mental illness includes questions about depression, anxiety, schizophrenia or alcoholism in the participant's mother or father. Sexual abuse questions assessed experiences of various types of sexual abuse by different people (e.g. family members, friends or strangers). Non-sexual abuse includes questions that capture physical or emotional cruelty and neglect by either parent/guardian. It is important to note that although there may appear to be overlap between 'maternal lack of care' and 'emotional cruelty or neglect', the questions assessing the latter reflect neglect by either parent/guardian; not just the mother.

Generating a psychosocial adversity score

All adversity variables were binary (coded as '0' for not exposed and '1' for exposed) except for maternal lack of care and overprotection, which were both continuous scores.(14) Thus, a binary variable was derived to indicate sub-optimal maternal bonding maternal lack of care and overprotection. All binary variables were then

summed to produce a summary score of the number of adverse experiences each participant was exposed to during childhood (ranging from 0-7). The summary score was categorised as 0, 1, 2, 3+. Linearity of associations between the summary score and CVD risk factors was assessed using a likelihood ratio test to compare models with the score as a continuous variable to models with the score as a categorical variable with indicators. There was no evidence of a threshold effect (results available on request). Thus, the categorical summary score was included in the regression model as a linear term.

Table S1. Pearson's correlation coefficients of cognitive function measures (n=1178).

	Verbal fluency test	Logical memory test	Digit backwards test	Digit symbol coding test	Delayed logical memory test	Spot the word test
**Verbal fluency test**	1.00					
**Logical memory test**	0.24	1.00				
**Digit backwards test**	0.36	0.23	1.00			
**Digit symbol coding test**	0.22	0.16	0.17	1.00		
**Delayed logical memory test**	0.24	0.83	0.23	0.19	1.00	
**Spot the word test**	0.43	0.31	0.33	0.15	0.31	1.00

All variables are scaled in the same direction so that higher values reflect better performance.

Table S2. Difference in specific cognitive function test scores at mean age 51 years between participants who were categorised as having an external vs internal locus of control at two time-points (N=1178).

	Unadjusted		Adjusted for potential confounders*		Additionally adjusted for earlier locus of control score	
	Mean difference (95% CI)	P	Mean difference (95% CI)	P	Mean difference (95% CI)	P
**Logic memory**						
Locus of control at mean age 30 years	-0.05 (-0.07 to -0.03)	<0.001	-0.04 (-0.06 to -0.02)	<0.001	-	-
Locus of control at mean age 48 years	-0.05 (-0.06 to -0.03)	<0.001	-0.04 (-0.05 to -0.02)	<0.001	-0.02 (-0.04 -0.003)	0.02
**Digit backwards**						
Locus of control at mean age 30 years	-0.05 (-0.07 to -0.03)	<0.001	-0.04 (-0.06 to -0.02)	<0.001	-	-
Locus of control at mean age 48 years	-0.05 (-0.07 to -0.03)	<0.001	-0.04 (-0.06 to -0.02)	<0.001	-0.03 (-0.05 -0.01)	0.01
**Spot the word test**						
Locus of control at mean age 30 years	-0.06 (-0.07 to -0.05)	<0.001	-0.04 (-0.06 to -0.03)	<0.001	-	-
Locus of control at mean age 48 years	-0.06 (-0.07 to -0.04)	<0.001	-0.04 (-0.06 to -0.03)	<0.001	-0.03 (-0.04 -0.01)	<0.001
**Digit symbol test**						
Locus of control at mean age 30 years	-0.02 (-0.03 to -0.01)	<0.01	-0.02 (-0.04 to -0.01)	<0.01	-	-
Locus of control at mean age 48 years	-0.03 (-0.05 to -0.02)	<0.001	-0.04 (-0.05 to -0.02)	<0.001	-0.03 (-0.04 -0.01)	<0.001
**Verbal fluency**						
Locus of control at mean age 30 years	-0.05 (-0.07 to -0.03)	<0.001	-0.04 (-0.05 to -0.02)	<0.001	-	-
Locus of control at mean age 48 years	-0.07 (-0.08 to -0.05)	<0.001	-0.05 (-0.07 to -0.03)	<0.001	-0.04 (-0.06 -0.02)	<0.001
**Delayed logic memory**						
Locus of control at mean age 30 years	-0.05 (-0.07 -0.03)	<0.001	-0.04 (-0.06 -0.02)	<0.001	-	-
Locus of control at mean age 48 years	-0.05 (-0.07 -0.03)	<0.001	-0.04 (-0.06 -0.02)	<0.001	-0.03 (-0.05 -0.01)	<0.01

CI- confidence interval. Results are interpreted as the average difference in the cognitive test scores between external versus internal locus of control

*Adjusted for maternal educational attainment, head of household social class, ethnicity and age at outcome assessment

Table S3. Associations between change in locus of control from mean age 30 years to 48 years and each of the six cognitive function test scores at mean age 51 years (N=1178).

	Unadjusted		Adjusted for potential confounders*	
**Change in LoC**	**Mean difference in cognitive function score (95% CI)**	**P**	**Mean difference in cognitive function score (95% CI)**	**P**
**Logical memory**				
Internal to internal (reference group)	-	-	-	-
External to external	-0.07 (-0.10 to -0.05)	<0.001	-0.06 (-0.08 to -0.03)	<0.001
Internal to external	-0.03 (-0.06 to 0.003)	0.08	-0.02 (-0.05 to 0.008)	0.15
External to internal	-0.03 (-0.06 to -0.01)	0.01	-0.03 (-0.05 to -0.003)	0.03
**Digit backwards**				
Internal to internal (reference group)	-	-	-	-
External to external	-0.07 (-0.10 to -0.05)	<0.001	-0.06 (-0.09 to -0.04)	<0.001
Internal to external	-0.03 (-0.06 to 0.01)	0.10	-0.03 (-0.06 to 0.01)	0.13
External to internal	-0.04 (-0.07 to -0.009)	0.01	-0.03 (-0.06 to -0.004)	0.03
**Spot the word test**				
Internal to internal (reference group)	-	-	-	-
External to external	-0.09(-0.10 to -0.07)	<0.001	-0.06 (-0.08 to -0.05)	<0.001
Internal to external	-0.04 (-0.06 to -0.02)	<0.001	-0.04 (-0.06 to -0.01)	0.001
External to internal	-0.05 (-0.06 to -0.02)	<0.001	-0.03 (-0.05 to -0.02)	<0.001
**Digit symbol test**				
Internal to internal (reference group)	-	-	-	-
External to external	-0.04 (-0.06 to -0.03)	<0.001	-0.05 (-0.06 to -0.03)	<0.001
Internal to external	-0.01 (-0.04 to 0.01)	0.24	-0.01 (-0.03 to 0.009)	0.25
External to internal	0.002 (-0.02 to 0.02)	0.81	0.0004 (-0.02 to 0.02)	0.96
**Verbal fluency**				
Internal to internal (reference group)	-	-	-	-
External to external	-0.06 (-0.09 to -0.04)	<0.001	-0.06 (-0.09 to -0.04)	<0.001
Internal to external	-0.05 (-0.08 to -0.02)	<0.001	-0.05 (-0.08 to -0.02)	<0.01
External to internal	-0.03 (-0.05 to -0.0005)	<0.01	-0.03 (-0.05 to -0.0005)	0.05
**Delayed logical memory**				
Internal to internal (reference group)	-	-	-	-
External to external	-0.07 (-0.10 to -0.05)	<0.001	-0.06 (-0.08 to -0.04)	<0.001
Internal to external	-0.04 (0-.07 to -0.005)	0.02	-0.03 (-0.06 to -0.008)	0.05
External to internal	-0.03 (-0.06 to -0.003)	0.03	-0.02 (-0.05 to 0.004)	0.10

CI- confidence interval. Results are interpreted as the average difference in the cognitive test scores (scaled from 0-1) between each group compared to the reference group

*Adjusted for educational attainment, head of household social class, ethnicity and age at outcome assessment

Table S4. Comparing unadjusted associations in the main analysis sample (n=1178) to the larger sample of participants with a measure of LoC at the first-time point only (n=2241).

	Unadjusted associations in main analysis sample (n=1178)		Unadjusted associations in larger sample (n=2241)	
	**Beta (95% CI)**	**P**	**Beta (95% CI)**	**P**
**Locus of control score at mean age 30 years**	-0.08 (-0.10 to -0.06)	<0.001	-0.09 (-0.10 to -0.07)	<0.001

CI- confidence interval. Results are interpreted as the average difference in the composite cognitive function score per unit increase in the locus of control score

Table S5. Average difference in the composite cognitive function score at mean age 51 years between participants who were categorised as having an external vs internal locus of control at two time-points, additionally adjusting for exposure to psychosocial adversity as a potential confounder (N=929).

	Unadjusted		Adjusted for potential confounders*		Additionally adjusted for previous locus of control score	
	**Mean difference (95% CI)**	**P**	**Mean difference (95% CI)**	**P**	**Mean difference (95% CI)**	**P**
**External vs internal locus of control at mean age 30 years**	-0.23 (-0.31 to -0.16)	<0.001	-0.19 (-0.26 to -0.12)	<0.001	-	-
**External vs internal locus of control at mean age 48 years**	-0.30 (-0.37 to -0.22)	<0.001	-0.26 (-0.33 to -0.19)	<0.001	-0.20 (-0.28 to -0.12)	<0.001

CI- confidence interval. Results are interpreted as the average difference in the composite cognitive function score between external versus internal locus of control

*Adjusted for educational attainment, head of household social class, ethnicity, the psychosocial adversity score and age at outcome assessment
